# Analysis and Optimization of Contact Material Ablation under the Cumulative Effect of the Number of Breakings of OLTC

**DOI:** 10.3390/ma16186344

**Published:** 2023-09-21

**Authors:** Mingyu Zhang, Yaxiong Tan, Chi Yang, Jun Deng, Zhicheng Xie

**Affiliations:** 1 State Key Laboratory of Power Transmission Equipment & System Security and New Technology, Chongqing University, Chongqing 400044, China; mingyuzhang@cqu.edu.cn (M.Z.); 202211021024@cqu.edu.cn (C.Y.); 2 EHV Power Transmission Company of China Southern Power Grid, Guangzhou 510000, Chinazcxie@hust.edu.cn (Z.X.)

**Keywords:** OLTC, vacuum interrupter, contact ablation, cumulative interrupting effects, thermal field distribution, contact materials

## Abstract

Vacuum on-load tap-changers (OLTC) for converter transformers have a much higher number of breakings than conventional circuit breakers. Contact ablation after several breakings will affect the stability and life of the device. This paper establishes the electromagnetic thermal multi-physical field coupling model of the vacuum interrupter for OLTC based on the finite element analysis method. The thermal field distribution of normal and ablative contact materials during the breaking process was analyzed. The key parameters affecting the contact temperature under the cumulative number of breakings are analyzed and the optimized design is completed. The simulation results show that the contact surface reaches a maximum temperature of 1390 K at 8 ms. There is a significant increase in the area of the high-temperature area on the contact surface. The possibility of re-ignition of the interrupter is increased. Based on the judgment matrix method, the key influencing parameters of the contact temperature rise are analyzed. The final parameters are selected as follows: contact material—CuCr8 alloy, contact seat thickness—2 mm, contact thickness—10 mm, and contact diameter—40 mm.

## 1. Introduction

An on-load tap changer (OLTC) is a crucial component of converter transformers in high-voltage direct current transmission systems. It facilitates significant adjustments in DC voltage levels, thereby ensuring operational safety and stability [1,2,3]. Traditional on-load tap changers rely on transformer oil for arc extinction as the arcing generated during circuit interruption can lead to insulation oil carbonization [4,5]. In order to ensure the performance of insulating oil, continuous maintenance of the breaker is essential during its operational process.

In recent years, the advancement of vacuum technology has driven the application of vacuum on-load tap changers. The arcs generated by vacuum circuit breakers do not interact with the medium, effectively avoiding the phenomenon of mineral oil carbonization [6,7]. Compared to traditional circuit breakers, vacuum circuit breakers have a longer lifespan, resulting in a significant reduction in maintenance frequency for vacuum on-load tap changers [7,8]. The design of vacuum interrupters profoundly influences the switching performance and lifespan of the switch. Currently, there is a trend towards the miniaturization, cost-effectiveness, and high-voltage capabilities of vacuum interrupters [9,10,11].

The temperature rise generated during the switching process is a crucial factor influencing the lifespan and breaking performance of switch devices. Some studies have been conducted concerning the thermal field distribution and temperature rise in vacuum interrupters. The influence of contact pressure, materials, and external magnetic fields on the temperature rise of on-load tap changer contacts has been analyzed [12,13,14]. The number of operations of on-load tap changers is far higher than that of conventional circuit breakers. The cumulative effect of the number of breakings leads to ablation of metal contact materials within the vacuum interrupter, which can impact the breaking performance. After multiple breaking operations, the resistance and other microscopic parameters of the contact material have changed. Currently, there is limited research addressing the ablation of contact materials within the vacuum interrupter under a cumulative number of breakings for on-load tap changers. Moreover, there exists a dearth of optimization studies pertaining to contact materials and structural parameters for on-load tap changers under conditions involving multiple breaking sequences. The material of on-load tap changer contacts needs to possess the capability to withstand ablation, while also meeting the requirement for the number of breakings. The optimal chromium content in the contact material remains uncertain for OLTC as well.

This paper establishes an electromagnetic–thermal multi-physics coupled model for the vacuum interrupter used in OLTC through the application of finite element analysis. The study investigates the thermal field distribution of normal and ablative contacts. Furthermore, it examines the changes in contact material resulting from a cumulative number of breakings. Subsequently, an optimization design is executed. After multiple breaking operations, OLTC contacts exhibit pronounced ablation issues, with the area ratio of the ablated contact surface surpassing the melting point of copper witnessing a significant increase. This phenomenon heightens the likelihood of re-ignition within the interrupter chamber. An analysis of the melting characteristics of copper–chromium alloy reveals the impact of chromium content on contact thermal field distribution. While an increased chromium content enhances resistance against ablation, it simultaneously exacerbates the issue of excessive temperature rises. Employing the analytic hierarchy process, key parameters influencing contact temperature rise are evaluated. Based on this analysis, contacts with low chromium content are selected and an optimized contact design is implemented.

## 2. Thermal Field Distribution of OLTC Contact Materials

### 2.1. Electromagnetic–Thermal Coupled Simulation Model of the Vacuum Interrupter

**Figure 1:**
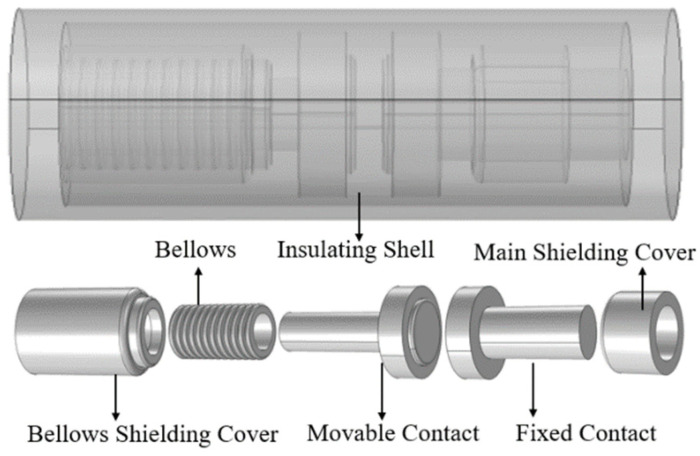
This is a figure. Schemes follow the same formatting.

Focusing on the vacuum-type OLTC employed in ±800 kV HVDC converter transformers, this study targets a rated voltage of 6 kV and a rated current of 1 kA. The three-dimensional simulation model is illustrated in Figure 1.

**Figure 2:**
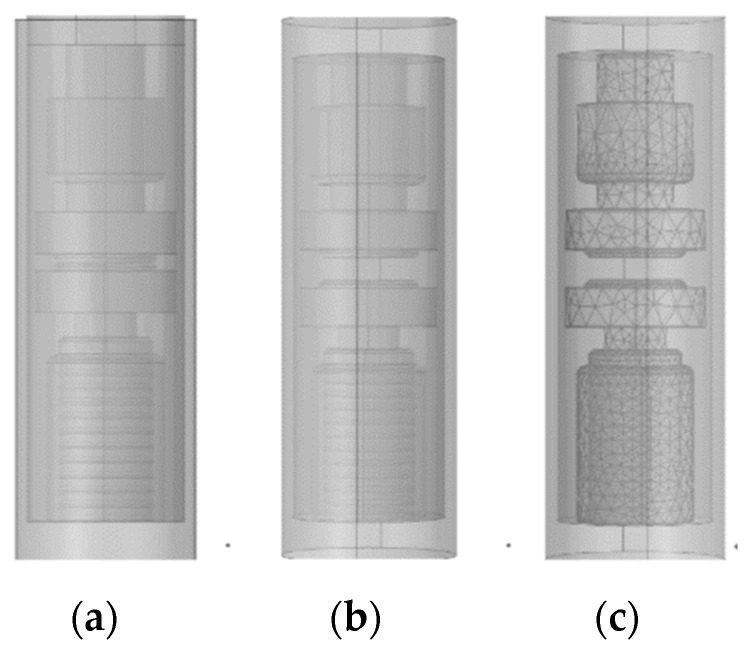
(**a**) Steady-state model, (**b**) transient model, and (**c**) meshing of the vacuum interrupter.

The mechanical strength of intricate contact structures is challenging to address, particularly considering that OLTCs are primarily designed to meet the breaking requirements of shifting operations, with limited current magnitudes. Consequently, the simulation model employs a disc-shaped contact with a 40 mm diameter and a contact gap of 5 mm. Based on the actual breaking conditions of the OLTC vacuum interrupter, both steady-state and transient models are constructed, as depicted in Figure 2.

The specific materials of internal components within the vacuum interrupter chamber are detailed in Table 1, with the interior being maintained under a vacuum condition.

In the range of 1 kA to 6.5 kA, the arc current of the vacuum arc exhibits a steady increase in arc voltage from 20 V to 40 V, with the columnar field strength increasing alongside the current augmentation [15,16]. Building upon this, an electromagnetic–thermal multiphysics coupled model is constructed to simulate the temperature rise within the vacuum interrupter chamber during the breaking process. A heat flux density is introduced as a boundary condition on the anode contact surface, simulating the heat generated by the breaking arc as a heat source, which heats the anode contact.

### 2.2. Steady-State Temperature Rise Characteristics

**Figure 3:**
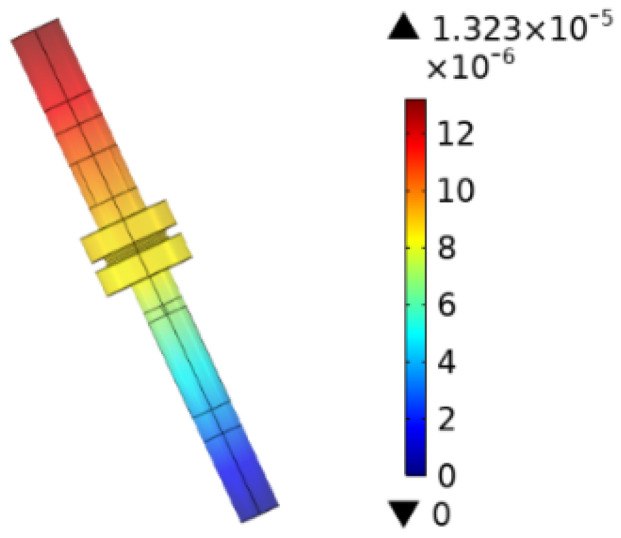
Contact potential through 1 A current.

The primary factor influencing the steady-state temperature rise is the circuit resistance of the interrupter chamber. This circuit resistance comprises the contact resistance on the contact surface and the metallic resistance of the moving and fixed contacts. Due to the irregular shape of the moving and fixed contacts, a conductor resistance validation model can be constructed for computation. By introducing a current module and allowing a steady-state simulation calculation with a current of 1 A passing between the moving and fixed contacts, the potential difference between the contacts represents the metallic contact resistance of the vacuum interrupter chamber. The simulation results are presented in Figure 3. From the simulated potential values between the metallic contacts, it can be deduced that the contact resistance R_1_ between the moving and fixed contacts is approximately 13 µΩ.

Calculating contact resistance requires considering both the contact pressure and the material of the contact. Currently, the mainstream calculation method involves using an empirical formula with added calibration coefficients. The calculation of contact resistance can be conducted using the following empirical formula:

(1) $$R_{f}=\frac{K}{(0.102F{)}^{m}}\times 10^{-3}$$

In the equation, *F* represents the contact pressure, *m* is the exponent dependent on the contact form, and, when the contact pressure is high, *m* typically falls within the range of 0.8 to 0.95. *K* is a coefficient related to factors such as the contact material and contact surface condition, with a value of 0.08 to 0.14 for copper–copper contacts.

The resistance value of the circuit resistance is equal to the sum of the contact resistance and the touch resistance:

(2) $$R=R_{1}+R_{f}$$

The calculated final value of the circuit resistance is 29 µΩ. In the preliminary research and experiments, we determined that the total resistance of the vacuum interrupter of the same model falls between 28 µΩ and 46 µΩ. The results obtained from the simulation model represent an idealized scenario. The contact surface is defined as smooth and there is no presence of oxides on the copper metal surface. Therefore, the resistance value is relatively small and it will be closer to the lower end of the resistance range. This result closely aligns with the computed contact resistance value.

**Figure 4:**
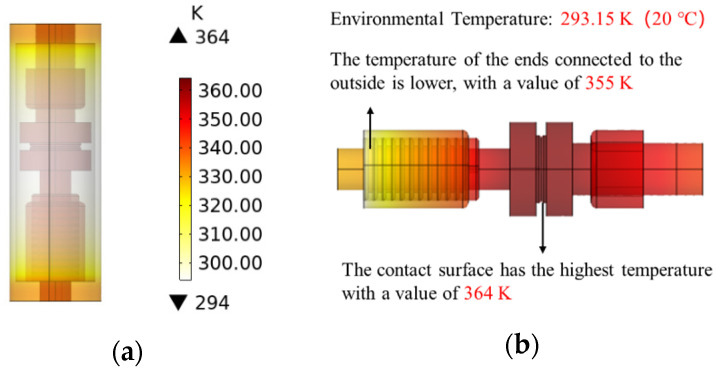
(**a**) Steady state heat field distribution of contacts through rated current; (**b**) Interrupter internal temperature rise.

At an ambient temperature of 293.15 K (20 °C), the thermal field distribution is depicted in Figure 4a.

The absence of convective heat dissipation within the vacuum interrupter results in temperature elevations being primarily influenced by heat conduction and thermal radiation. Observing Figure 4 reveals that the upper and lower ends of the interrupter chamber shell experience higher temperature rises. This phenomenon arises from the conduction of heat from the contacts and shielding cover towards both ends, resulting in elevated temperatures at the contact locations. Due to inferior thermal conductivity, the central portion of the interrupter chamber shell exhibits a lower temperature rise, approximately around 5 K. The internal temperature rise within the interrupter chamber is illustrated in Figure 4b.

The temperature elevation across different internal components within the interrupter chamber is presented in Table 2.

Upon observation, it is evident that the highest temperature rise occurs at the interface between the moving and fixed contacts, reaching a value of 70.95 K. This phenomenon is attributed to the presence of contact resistance at the said interface. The convective heat transfer at the outer region of the interrupter chamber contributes to the dissipation of a portion of the generated heat, resulting in lower temperature rises at both ends.

### 2.3. Thermal Distribution during the Interruption Process

The simulation model employs the method of adding heat flux density to the anode contact surface to simulate the continuous heating process of the arc on the anode contact in the vacuum interrupter chamber [17,18,19,20]. The mathematical expression for the heat flux density is as follows:

(3) $$q=H_{p}e^{-5000^{r^{2}}}\sin\left(100\pi t\right)$$

**Figure 5:**
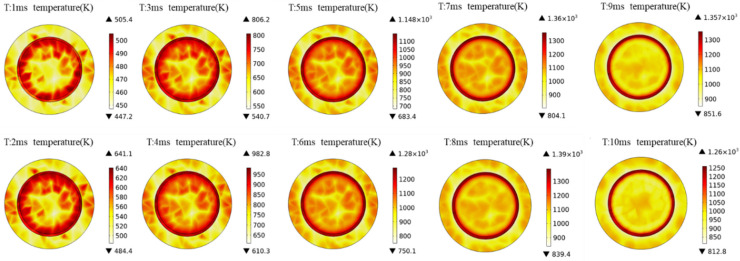
Temperature distribution of anode contacts at different times.

In the equation, *H_p_* represents the peak heat flux density and *r* stands for the contact gap distance. The alternating current vacuum arc generally extinguishes during the zero-current crossing, with an arc-burning time equivalent to half a cycle of the power frequency. Transient processes occur based on a steady-state condition. Therefore, the steady-state temperature rise on the contact surfaces is set as the initial value. The thermal simulation results for the anode contact of the vacuum interrupter during the opening process are illustrated in Figure 5.

**Figure 6:**
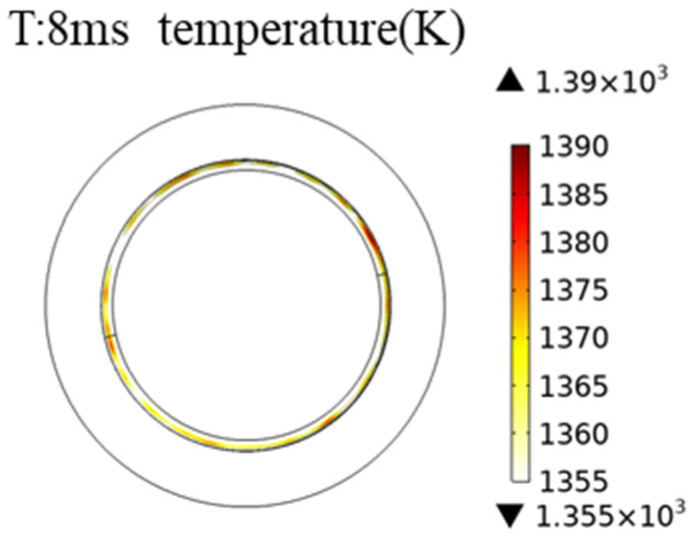
High-temperature area on the contact surface at 8 ms (above 1355 K).

It can be observed that during the opening of the switch, the temperature of the anode contact in the vacuum interrupter reaches its peak at 8 ms, reaching a maximum temperature of 1390 K. The simulated contact material is pure copper and the highest temperature of the anode contact during the arc-extinction process exceeds the melting point of copper (1355 K). By analyzing the proportion of the contact surface area exceeding the melting point of copper at each moment, it is observed that at 8 ms, the proportion of the contact surface area exceeding the copper melting point is 6.4%, as illustrated in Figure 6.

**Figure 7:**
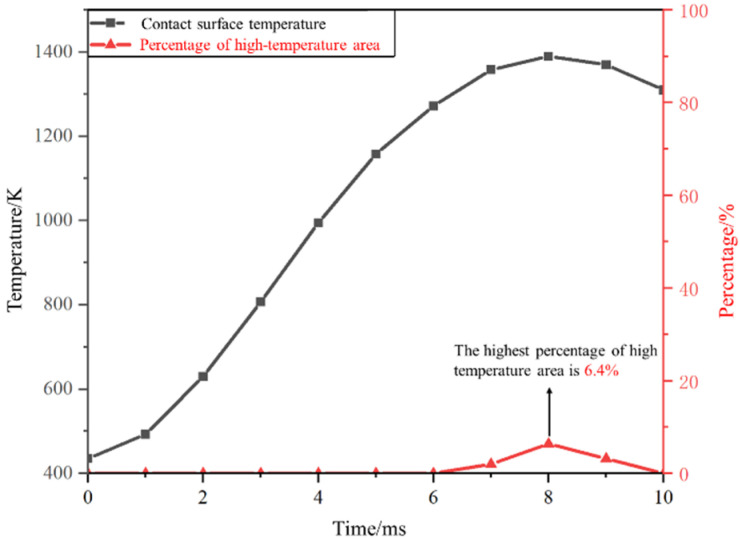
Curve of the maximum temperature with time during an interruption.

The variation in the percentage of the highest temperature rises and the high-temperature region area is depicted in Figure 7.

The arc voltage of alternating current vacuum arcs changes with the variation in sinusoidal current. By observing the changing curve, it is evident that the anode contact temperature rises rapidly within the 2–4 ms period, during which the arc current gradually increases to its peak. Following the peak current, the arc voltage decreases and the temperature rise of the anode contact slows down. Between 9–10 ms, as the AC current approaches zero crossing, the anode contact temperature also exhibits a decreasing trend, ultimately reaching around 1260 K. An area exceeding the melting point of copper is observed on the contact surface between 7–9 ms. During the interruption process, it is normal for a portion of the vacuum interrupter’s contact surface temperature to exceed the metal melting point, resulting in slight ablation of the contact material. This phenomenon does not lead to a significant amount of metal vapor residue within the gap. Examining the change in the proportion of the high-temperature region reveals that the area exceeding the copper melting point is relatively small. Thus, the temperature rise during this phase does not affect the normal interruption process of the OLTC.

Analyzing the thermal distribution depicted in Figure 6, it is apparent that the highest temperature is in the circular ring section with a radius of approximately 0.15 mm on the surface of the OLTC flat contact. This phenomenon is attributed to the structure of the anode contact. Different structures of contacts made from the same material exhibit distinct temperature rise patterns under the influence of arc heat sources. This aspect underscores the need for careful consideration when employing complex contact structures. Areas with higher contact surface temperatures are more prone to severe ablation. Consequently, the subsequent ablation model for the contacts will simulate the ablation locations based on the thermal distribution observed in the simulation results.

## 3. Influence of Accumulated Number of Breakings on Different Contact Materials

**Figure 8:**
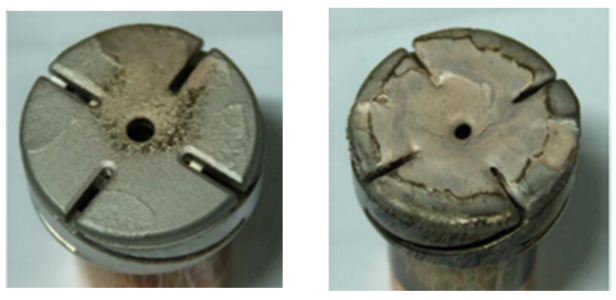
Ablation of contact surface under cumulative shift times.

Under the influence of the vacuum arc current, the surface temperature of the anode increases, surpassing the melting point of the contact material. Figure 8 illustrates the surface ablation condition of the contact.

### 3.1. Analysis of Ablation in Copper Contacts

After multiple breaking operations, the roughness of the contact surface increases, leading to an elevation in the contact resistance value. Since the contact resistance constitutes a significant proportion of the circuit resistance within the interrupter chamber, the temperature rise in OLTC is significantly influenced. Contact resistance is composed of both shrinkage resistance and surface film resistance, with the value of the surface film resistance experiencing an increase [21,22]. The formula for calculating the surface film resistivity is as follows:

(4) $$\sigma_{F}=\rho_{F}\cdot d$$

Based on the surface film growth curve of copper contacts at corresponding temperatures, the thickness of the film (*d*) can be determined and the surface film resistivity ($\rho_{F}$) can be obtained from the curve relating film thickness to resistivity. The formula for calculating surface film resistivity is as follows:

(5) $$R_{f}=\frac{\sigma_{F}}{\pi a^{2}}$$

In the equation, *a* represents the effective contact radius and $R_{f}$ stands for surface film resistance. After calculating the surface film resistance, the contact resistance for different numbers of breakings is estimated using an empirical formula, as presented in Table 3.

**Figure 9:**
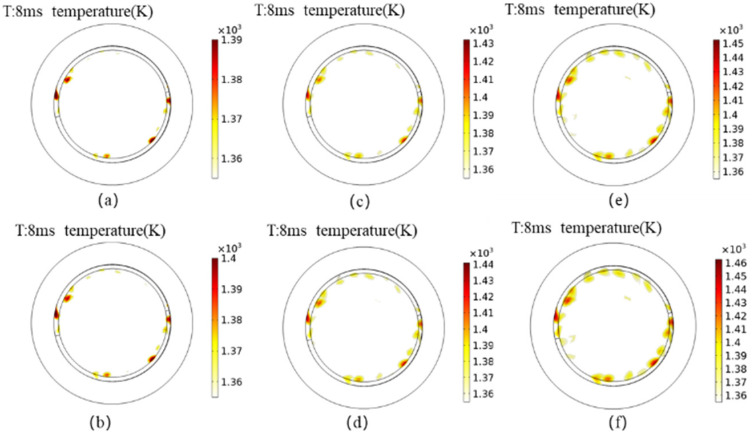
Break times’ cumulative effect on the high temperature area; (**a**) Intact contacts; (**b**) 20,000 openings; (**c**) 40,000 openings; (**d**) 60,000 openings; (**e**) 80,000 openings; (**f**) 100,000 openings.

The thermal distribution of the high-temperature region on the contact surface (exceeding the copper melting point of 1355 K) for different numbers of breakings is illustrated in Figure 9. Upon observation, it is evident that with 100,000 breakings, both the highest temperature on the contact surface and the proportion of the high-temperature region have notably increased. To contrast and analyze the cumulative effect of the number of breakings on contact ablation, the temperature rises within 10 ms for both the pristine and 100,000 breakings scenarios are extracted and plotted in Figure 9.

After 100,000 breakings, the contact resistance is increased by approximately 15%. At this point, the highest temperature reached on the contact surface i1465 K, representing a 5% increase compared to the pristine contact. Furthermore, the area proportion of the contact surface exceeding the copper melting point has reached 14.5%, exacerbating the ablation situation. The occurrence of noticeable ablation after 100,000 breakings demonstrates the progressive degradation of the contact. The elevated temperature and surface roughness resulting from ablation will further escalate the ablation process, leading to more severe ablation-related issues.

**Figure 10:**
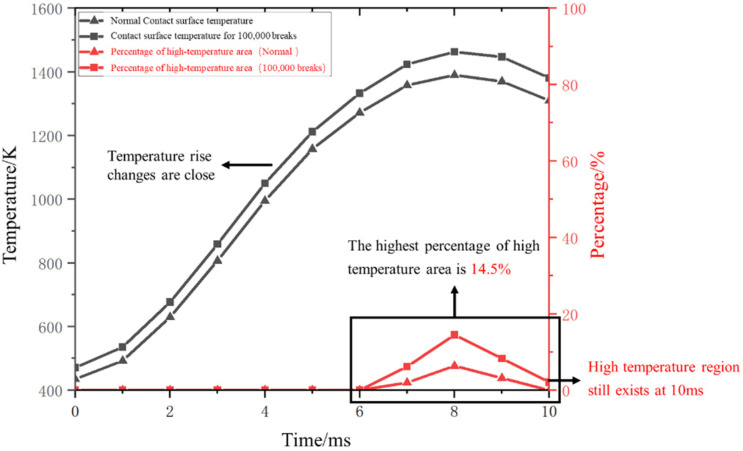
Temperature change after several open breaks with high temperature area percentage.

As depicted in Figure 10, at the moment of current zero crossing (10 ms), there is a decrease in the temperature of the contact surface.

However, regions with temperatures exceeding the copper melting point persist. During this time, the molten region on the contact surface continues to release a significant amount of metallic vapor into the gap. As the recovery voltage rises, there is a possibility of restriking breakdown, leading to arc re-ignition, and ultimately leading to failure of the switch opening.

### 3.2. Temperature Rise Analysis of Different Copper–Chromium Alloy Contacts

The choice of contact material is a crucial factor influencing circuit breaker performance and copper–chromium alloy contacts have demonstrated excellent breaking capabilities. The key determinant of copper–chromium alloy contact performance is the chromium content. The requirements for OLTC differ from those of conventional circuit breakers. To identify a more suitable contact material for OLTC applications, a comparative analysis of the temperature rise in copper–chromium alloy materials with varying chromium contents was conducted.

**Figure 11:**
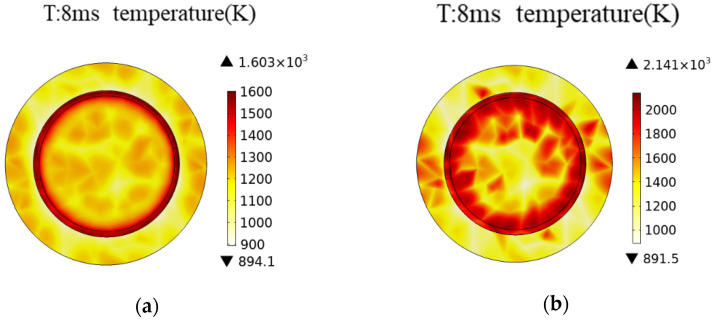
Distribution of the thermal field in anode contacts of different contact materials. (**a**) CuCr8; (**b**) CuCr26.

The transient temperature rise following the replacement of contact material is illustrated in Figure 11.

Upon changing the material, the surface temperature of the anode contact within the vacuum interrupter significantly surpasses the temperature observed with pure copper contacts. This difference is attributed to the lower electrical conductivity of copper–chromium alloy in comparison to pure copper. Consequently, when breaking the same magnitude of current, the temperature rise of the contact is higher. The results of the simulation experiments align with the anticipated outcomes. Copper has a significantly lower melting point (1355 K) than chromium (2130 K), resulting in distinct melting behaviors between copper–chromium alloys and pure metallic materials. On the surface of the copper–chromium alloy contact head, distinct regions of complete melting and partial melting manifest, aligning directly with the actual temperature distribution. At elevated temperature zones (exceeding the melting point of chromium), both copper and chromium undergo the process of melting. Within the partially molten region, copper undergoes melting while chromium remains in its solid state, leading to the formation of a pasty region [23,24,25]. The simulation results validate the melting characteristics of copper–chromium alloys.

**Figure 12:**
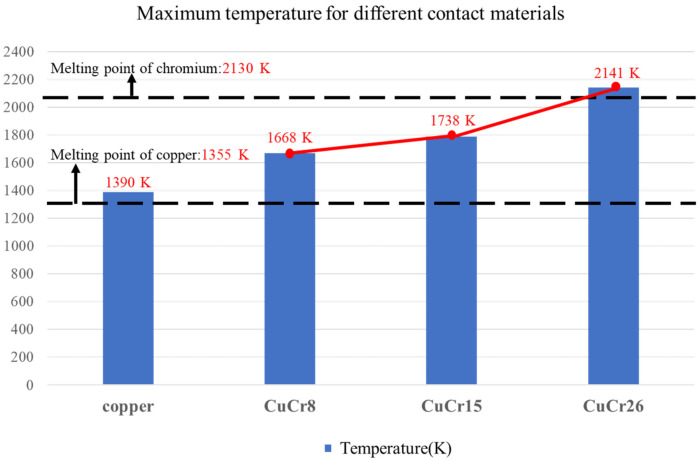
Comparison of the temperature rise of different contact materials.

As shown in Figure 12, the maximum surface temperatures for the CuCr8 and CuCr15 contacts are 1668 K and 1738 K, respectively.

These temperatures do not reach the melting point of chromium, thus preventing the occurrence of a complete melting region. In contrast, the CuCr26 contact exhibits a maximum temperature rise of 2141 K, attributed to the increase in the content of metallic chromium, which subsequently leads to an elevation in the resistance value of the contact. Contacts with lower chromium content only generate partial melting zones; the resulting generation of metallic vapor and the ablation status of the contact remain within controllable limits. At a breaking current of 1 kA, the contact will not experience severe breakdown phenomena. Considering the operational requirements for the electrical lifespan of on-load tap changers, copper–chromium alloy contacts demonstrate a relatively minor ablation situation compared to those made of pure copper. However, the determination of the appropriate metallic chromium content still requires further research.

## 4. Multi-Factor Optimization Design of OLTC Contacts

**Figure 13:**
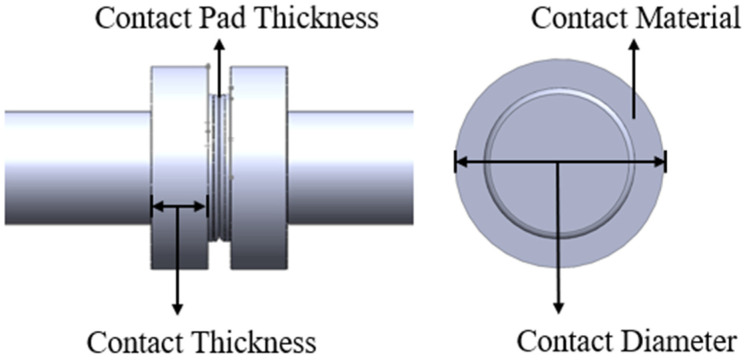
Parameters affecting contact temperature rise.

Based on the preceding study, four parameters that influence the temperature rise of contacts in on-load tap changer vacuum interrupter chambers have been identified: contact material (A), contact thickness (B), contact pad thickness (C), and contact diameter (D), as depicted in Figure 13.

The analytic hierarchy process (AHP) method was employed to determine the relative importance of different parameters and achieve a multi-factor optimization design of contacts in a vacuum interrupter [26]. For the purpose of comparison, each parameter was normalized, resulting in the factor level coding Table 4 as shown below.

**Figure 14:**
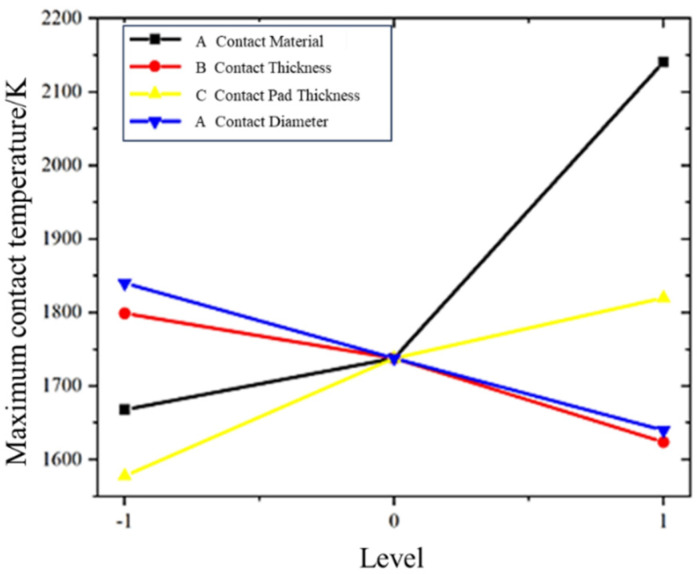
Distribution of the thermal field in anode contacts of different contact materials.

Initially, the weight coefficients of the parameters influencing temperature rise were determined. Referring to the relationship between contact temperature rise and the four design parameters in Figure 14, an initial judgment matrix was constructed.

The matrix coefficients represent the relative importance of different objectives. The final judgment matrix A is as follows:

$$\left[\begin{array}{cccc} 1 & 3.63 & 2.11 & 4\\ 0.275 & 1 & 0.58 & 1.1\\ 0.48 & 1.724 & 1 & 1.9\\ 0.25 & 0.91 & 0.526 & 1 \end{array}\right]$$

By further calculation, the importance parameter for each individual parameter can be obtained.

(6) $$a_{i}=\sqrt[{4}]{a_{i1}a_{i2}a_{i3}a_{i4}}$$

In the equation, $a_{i1}$ represents the corresponding coefficient in the matrix. After normalization, the weight coefficients *ω_i_* of the objective functions can be obtained:

(7) $$\omega_{i}=\frac{a_{i}}{\sum_{j=1}^{4}a_{j}}$$

The final weight coefficient vector for the key parameters of the vacuum interrupter contact temperature rise in the on-load tap changer is obtained as follows:

$$W={\left[0.4997,0.1374,0.2379,0.1250\right]}^{T}$$

Upon observing the weight coefficient vector, it is evident that the contact material and contact seat thickness hold significant influence on the temperature rise, thus warranting prioritized consideration during the design process. Considering the electrical endurance requirements of on-load tap changers, where contacts do not need to interrupt high currents, the selection of CuCr8 as the contact material is appropriate. This choice enhances anti-ablation capability while avoiding excessive temperature rises. A contact seat thickness of 2 mm is also selected for the same reasons. The impact of contact diameter and contact thickness on the contact temperature rise in the vacuum interrupter is relatively minor. Increasing these dimensions can marginally decrease the temperature rise. However, this comes with the trade-off of enlarging the interrupter’s size, which complicates device arrangement and contradicts the compactness required for on-load tap changers. While increasing the contact diameter can effectively enhance breaking capacity, in the context of on-load tap changers, where contacts do not need to handle high current interruptions, prioritizing electrical endurance and anti-ablation capability is more crucial. Consequently, the optimized design parameters for the vacuum interrupter are as follows: contact material—CuCr8 alloy, contact seat thickness—2 mm, contact thickness—10 mm, and contact diameter—40 mm.

## 5. Conclusions

This study has established a coupled electromagnetic–thermal multiphysics simulation model for the vacuum interrupter of OLTC. The research focused on the thermal distribution patterns during contact opening and the effects of contact material variations. Moreover, the investigation analyzed crucial parameters impacting contact temperature rises under cumulative breaking cycles and conducted optimization design. The conclusions drawn from this study are as follows.

Utilizing finite element analysis, this study investigated the steady-state and transient thermal distribution patterns of contact materials in the vacuum interrupter of OLTC. For copper contacts, the steady-state maximum temperature reached around 364 K. In transient conditions, contact with a 40 mm diameter exhibited a peak temperature of approximately 1390 K when breaking 1 kA current, surpassing the melting point of copper (1355 K). The high-temperature area occupied about 6.4% of the contact surface. The highest temperature was attained during the 8 ms arc duration. The structure and material of the contact had substantial influence on the thermal distribution during the breaking process, with the anode contact showing the highest temperature around a circular area with a radius of approximately 15 mm.

The impact of ablation on different contact materials was analyzed. Under ablation conditions, the contact resistance of copper contacts increased, leading to a significant temperature rise compared to intact contacts. The area of the ablated contact surface exceeding the melting point of copper accounted for 14.5%, higher than the 6.4% in normal contacts, thereby elevating the possibility of re-ignition within the interrupter. Combining this with the analysis of the melting characteristics of copper–chromium alloys, the influence of chromium content on contact thermal distribution was examined. Consequently, a contact material with lower chromium content was selected based on the operational conditions of the OLTC.

Utilizing the analytic hierarchy process, the weighted factors influencing the contact temperature rise were analyzed and an optimized contact design was achieved. The prioritized influence of each parameter on the contact temperature rise was as follows: contact material > contact seat thickness > contact thickness > contact diameter. Consequently, the optimized design parameters for the interrupter chamber were determined as follows: contact material—CuCr8 alloy, contact seat thickness—2 mm, contact thickness—10 mm, and contact diameter—40 mm.

## Figures and Tables

**Table 1:** Material properties of the vacuum interrupter simulation model.

Interrupter Structure	Title 2	Title 3
Contacts	Copper	5.8 × 10^7^
Insulating Shell	Ceramics	10^−8^
Bellows Shielding Cover	Stainless Steels	-
Main Shielding Cover	Stainless Steels	-
Bellows	Stainless Steels	-

**Table 2:** Vacuum interrupter temperature rise.

Interrupter Structure	Maximum Temperature Rise/K	Highest Temperature/K
Anode Contacts	70.95	364.10
Cathode Contacts	70.81	363.96
Insulating Shell	37.44	330.59
Bellows Shielding Cover	65.10	358.16
Main Shielding Cover	69.02	362.17
Bellows	67.28	360.43

**Table 3:** Variation in on-load tap-changer contact resistance with the number of breaking operations.

Number of Breakings/Thousand	Contact Resistance/μΩ	Total Resistor/μΩ
0	16.02	29.02
20	16.54	29.54
40	17.16	30.16
60	17.87	30.87
80	19.08	32.08
100	21.35	34.35

**Table 4:** Level code table of factors affecting contact temperature rise.

Level	A	B/mm	C/mm	D/mm
−1	CuCr8	5	2	35
0	CuCr15	10	4	40
1	CuCr26	15	6	45

## Data Availability

Not applicable.
